# When Contact Is Not Enough: Affecting First Year Medical Students’ Image towards Older Persons

**DOI:** 10.1371/journal.pone.0169977

**Published:** 2017-01-20

**Authors:** Sasmita Kusumastuti, Esther van Fenema, Eugenie C. F. Polman-van Stratum, Wilco Achterberg, Jolanda Lindenberg, Rudi G. J. Westendorp

**Affiliations:** 1 Department of Public Health, and Center for Healthy Aging University of Copenhagen, Copenhagen, Denmark; 2 Department of Psychiatry, Leiden University Medical Centre, Leiden, the Netherlands; 3 Leyden Academy on Vitality and Ageing, Leiden, the Netherlands; 4 Department of Public Health and Primary Care, Leiden University Medical Centre, Leiden, the Netherlands; University of Brescia, ITALY

## Abstract

**Context:**

Many medical schools have initiated care internships to familiarize their students with older persons and to instil a professional attitude.

**Objective:**

To examine the impact of care internships on the image that first-year medical students have of older persons and to explore the underlying concepts that may play a role in shaping this image.

**Design:**

Survey before and after a two-week compulsory care internship using the Aging Semantic Differential (ASD; 32 adjectives) and the Attitudes toward Old People (AOP; 34 positions) questionnaires.

**Participants:**

Before and after a care internship involving interpersonal contact, 252 and 244 first-year medical students at the Leiden University Medical Centre (LUMC) in the academic year 2012–2013 participated.

**Method:**

Descriptive statistics, analyses of variance, and principal component analysis were used; clusters of adjectives and positions were reduced into concepts to examine dominant patterns of views. Changes in image were investigated as mean differences of the total and concept scores.

**Results:**

Both the ASD and the AOP questionnaires showed a poor general image of older persons that significantly worsened after the care internship (p < 0.01). The percentage of students considering over 75 years as being old increased from 17.2% to 31.2% (p < 0.01) and those who thought they would find as much satisfaction in care for older as for younger patients decreased from 78.5% to 62.1% (p < 0.001). Exploratory principal component analysis showed particularly low scores on ‘comportment’ and ‘pleasurable interaction’ whereas the scores on ‘personality traits’ and ‘habitual behaviour’ significantly deteriorated (both p < 0.001). These patterns were irrespective of the student’s gender and previous contact experience.

**Conclusion:**

Medical schools should carefully consider care internships to ensure that students do not worsen their views on older patients, which may occur due to inadequate contact depth and quality within a rather unsupportive context.

## Introduction

Current medical students might not be fully aware that their future professional career will mostly revolve around older people. After all, older persons have become a significant proportion of the world’s population and it is estimated that this proportion will steadily increase[1]. Older persons will be the majority of patients they have to take care of, as age is the most significant risk factor of functional decline, disease, and disability[2]. This demographic change presents a plethora of challenges; the increasing demand for professional and informal caregivers being one of them[3]. Although ageing is a consequential part of life, older persons are often viewed in a negative light[4–6]. Ageism–stereotypes of and prejudice against older persons—can inadvertently affect health professionals when deciding their future career paths. In fact, despite that older persons already make up the largest part of their patients, most first year medical students do not consider the geriatric field as a likely choice[7].

People, medical professionals included, commonly use age next to sex and race to categorize others. Our own fear of becoming frail, dependent, and to die may be the reason ageism is widespread, persistent, and found cross-culturally[8]. In an effort to get students acquainted with and interested in the older population, many medical schools have initiated care internships for their future physicians. These efforts can be seen as ways to make the unfamiliar familiar and line up with the intergroup contact hypothesis[9]. Deliberate interpersonal contact has long been one of the most influential strategies for improving relations between groups that are unfamiliar with each other[10] and an effective way to reduce prejudice between majority and minority group members[11]. Scholars have proposed that such a positive effect may only be expected under appropriate conditions[12,13] including sufficient knowledge, equal status between groups, interdependent goals, intergroup cooperation, personal and positive contact, an instilment of the positive experience in attitudes towards the other group, and the support of the involved institutions and authorities [9,14].

It is to address this triad of appropriate knowledge, contact, and attitude, and the effects of these on the image of young medical professionals in training why many medical schools have initiated care internships. These usually aim to familiarize their students with frail older persons and instil the necessary professional behaviour. It is yet unclear how a care internship influences young medical professional’s perception of older persons. On the one hand, it is possible that it contributes to a positive image of young towards older. On the other hand, it may not fulfil all necessary conditions and conversely instil a negative image. Here we report on the effects of a two-week compulsory care internship for first-year medical students emphasising interpersonal contact. It is of vital interest to examine the impact of care internships on the image young future medical professionals hold towards older people and to discover underlying concepts shaping this image, as the image created during this experience may significantly influence their training and eventually their career choices. To do so, we conducted a thorough pre- and post-survey study consisting of questionnaires comprising statements and series of opposing concepts regarding older persons.

## Materials and Methods

### Ethics statement

The Leiden University Medical Center Medical Committee Medical Ethics (CME) reviewed our protocol and decided that it did not have any objection to the study and we proceeded according to protocol. This "no objection" was received under the number P12.157/NV/nv.

Participants were asked to voluntarily participate in the study and could decline at any time. Students were informed of the purpose of the study in a letter that was attached to the questionnaires. In this letter they were explicitly informed about the purpose of the study, that the study was anonymous and did not have any impact on their study results. According to protocol and with no objection of the Leiden University Medical Center Medical Committee Medical Ethics verbal, instead of written, consent was given when handing out the form. Written consent was not obtained as to avoid any impression that choosing not to fill in the questionnaire would have effect on the study outcomes for the students declining participation. For the minors involved in this study, we followed the same procedure.

### Study population

The study population consisted of all 315 first-year medical students at the Leiden University Medical Centre in the academic year 2012–2013.

### Intergroup contact

The intergroup contact took place in the form of a two-week compulsory care internship to gain experience in working with care-dependent older patients. The majority of the students (78.7%) were assigned to a nursing home, a rehabilitation centre, or a psychogeriatric ward. The rest were assigned to home care, screening departments for rehabilitation, and specialized departments in hospitals. For most of the first-year medical students, this care internship is their first professional encounter with patient care. The students have a common curriculum and learning outcomes. This internship is to expose the students to care dependent older patients and to learn to reflect on the student’s own fears and emotions regarding sickness, multi-morbidity, dependency, declining quality of life, death, and also on cultural differences. Further, they have the opportunity to reflect on their own motivation for their study and future profession. The care internship has a study equivalent of ± 105–120 hours, excluding reporting. The students have a supervisor at their host institute to guide and help them achieve their learning outcomes, team managers and nurses supervised most of them. The tasks that the students practice during the care internship are as follows:

- Washing, dressing, and grooming of patient’s appearance;
- Transferring patients to help themselves and toileting;
- Help with eating, drinking, and taking medication;
- Guiding daily activities of a patient;
- Conduct a professional interview with a patient about complaints and problems.

### Procedure

At the beginning of the academic year, before and after the care internship, first year medical students were asked to voluntarily and anonymously fill in the survey. The first survey was conducted on 6 September 2012 prior to an introductory seminar on geriatrics, to eliminate influence caused by the preparatory education. The survey was distributed with a cover letter and additional oral explanation in the lecture hall. After filling in the survey, the students were given a seminar that included an introductory lecture along with an assignment and a movie about an older person. The second survey was taken immediately after their care internship in a concluding seminar on 15 October 2012, also accompanied by written and oral explanation. Both pre- and post-internship surveys were then anonymously and digitally processed.

### Questionnaires

The survey was based on three questionnaires, one being a demographic characteristics questionnaire that also asked about previous contact with older persons (see S1 Appendix). The two other questionnaires were the Aging Semantic Differential scale and a translated version of the Kogan scale[15] to measure attitudes towards older persons. The Aging Semantic Differential is a 7-point scale consisting of dichotomies at either end of the scale, containing 32 adjectives[16]. The Kogan scale questionnaire, labelled here as Attitudes toward Old People, consists of 34 positions, 17 positive, 17 negative, Likert scale type answers[17]. Earlier research in Dutch with these questionnaires by Beullens and Martens found a Cronbach's Alpha of 0.87 for the translated Aging Semantic Differential scale and 0.74 for the Kogan scale[18]. Both questionnaires seem sufficiently sensitive in the Dutch context.

### Statistical analysis

Data was screened for univariate outliers; 9 out-of-range values were identified and recoded as missing data due to administrative errors. Demographics characteristics were described, with missing items included in the calculation of proportion.

Questionnaires used in this study consist of statements and series of opposing concepts regarding older persons. Participants were asked to rate each statement and concept on a 7-level Likert scale, for example ranging from ‘strongly disagree’ to ‘strongly agree’. Scores were calculated for each item on a scale of 1 to 7, the latter indicating worst possible image. The Aging Semantic Differential has 32 adjectives and the Attitudes toward Old People has 34 positions, amounting to the worst possible image total score of 224 and 238 points respectively. The questionnaires were examined on a population level and were not evaluated on the individual level.

Our analysis consisted of two steps. First, an independent t-test was performed on the total scores of each questionnaire to analyse general image as perceived by students before and after intergroup contact. Second, to better interpret the large number of variables measuring different aspects of older persons, an exploratory Principal Component Analysis (PCA) was conducted to discover if the original variables reflected patterns that could explain the variance in the data. Based on these original variables, PCA helps analyse the underlying structure in the data by constructing new characteristics that best summarize the many variables. Hence, PCA helps extract the most relevant patterns in the data in a concise manner to better understand the results. The suitability of the dataset was assessed for the basis of PCA. For the two questionnaires used, the Kaiser-Meyer-Olkin measure of sampling adequacy was 0.87 and 0.76 respectively, above the recommended value of 0.6, and Bartlett’s tests of sphericity were significant for both (p < 0.001), indicating that there are correlations in the dataset that are appropriate for PCA. Principal component analyses were conducted with oblique rotation (see S2 Table and S3 Table for component loadings). Clusters of adjectives and positions were identified to measure underlying dimensions and were reduced into concepts that allowed for examining the dominant patterns of views in the data. Changes in image were measured in mean differences of the total and concept scores before and after the care internship.

## Results

Before and after the care internship, 252 and 244 students filled in the survey amounting to a response rate of 80% and 77% respectively.

### Demographic characteristics

After eliminating outliers, data before the internship was collected from 248 first year medical students (see Table 1). The majority of students were 18 years old (42.7%), with the number of females almost twice the number of male participants (65.3% and 33.5%). Only a few students (1.6%) indicated living with an older person. Most of them had at least one grandparent still alive (89.9%) and the majority (95.2%) did not have a living great-grandparent anymore. As for experience, most students did not have previous experience in caring for older persons through an internship (82.7%), working (69.8%), or volunteering with older persons (80.6%). Most of them (71.8%) did not have experience as informal caregiver, but a third of the population had someone in the family who was an informal caregiver (31.5%). 60.5% preferred to work in a hospital during the internship. After the care internship, 239 students completed the same set of questionnaires; demographic characteristics of this sample not being significantly different from the sample of 248 students presented in Table 1.

**Table 1:** Demographic Characteristics of the Students before the Care Internship.

Demographic Characteristics	N = 248		%	
Age (years)	<17	11.3
	18	42.7
	19	24.6
	20	14.1
	21	2.4
	22	1.6
	>23	2.0
Gender	Female	65.3
Living in a house with older person	Yes	1.6
Family member / friend / neighbour with dementia	Yes	31.5
Number of grandparents still alive	0	9.3
	1	18.1
	2	35.5
	3	24.2
	4	12.1
Number of great grandparents still alive	0	95.2
	1	2.8
	2	0.8
Previous internship with older persons	Yes	16.9
Working experience with older persons	Yes	28.2
Volunteer experience with older persons	Yes	16.5
Experience working as caregiver	Yes	19.0
If experienced, as a caregiver for	Friend	1.6
	Nuclear family	4.8
	Neighbours	1.6
	Extended family	12.1
Someone in the family gives care	Yes	31.5
Institution preferred for internship	Residential care facility	10.1
	Nursing home	7.7
	Home care	4.0
	Hospital	60.5
	Psychiatry	13.3

### Change in general image

Total scores from before and after questionnaires were compared to assess change in general image, with higher scores indicating worse image towards older people. As seen in Table 2, we found that before contact, on average students scored 118.4 ± 16.5 out of 224 for Aging Semantic Differential and 112.1 ± 19.4 out of 238 for Attitudes towards Old People, showing poor image to begin with. After contact, students scored significantly higher on the total scores, indicating a worsening general image on both questionnaires (122.7 ± 18.9 for Aging Semantic Differential and 118.4 ± 17.4 for Attitudes toward Old People) compared to before contact. Adjusting for the student’s gender and previous contact experience did not alter our findings.

**Table 2:** Effect of Intergroup Contact on General Image.

Questionnaire	Full Range of Scores	Change in Mean (SD)		Mean Difference (SE)
		Before Contact	After Contact	
Aging Semantic Differential	0–224	118.4 (16.5)	122.7 (18.9)	4.2 (1.6)*
Attitudes toward Old People	0–238	112.1 (19.4)	118.4 (17.4)	6.3 (1.7)**

Higher scores indicate worsening general image after intergroup contact.

Independent t-test analysis * p < 0.01, ** p < 0.001.

Table 3 shows the image as perceived by students before and after the internship. After this intergroup contact, views on which people to consider old had shifted significantly to a higher chronological age with the percentage of students considering over 75 years as old increasing from 17.2% to 31.2% (p < 0.01). After contact, the number of students who thought they would find as much satisfaction in care for older patients as for younger patients significantly decreased from 78.5% to 62.1% (p < 0.001). Although most students still answered that they would find as much satisfaction in care of older people as with younger patients, only 0.8% would certainly want to work with older persons, and the majority of them, 60.6%, would rather not and 6.2% would certainly not work with patients older than 65 years. After contact, there were no significant changes in these proportions.

**Table 3:** Comparison of General Image Before and After Intergroup Contact.

		Before	After
		Contact	Contact
		%	%
Age group considered as old (years) *	>55	1.6	1.3
	>60	8.2	5.6
	>65	42.2	41.0
	>70	30.7	20.9
	>75	16.0	27.4
	>80	1.2	3.8
Will find as much satisfaction in care of older patients as with younger patients **	Yes	78.5	62.1
Would like to work in a practice where most patients are >65 years after training	Certainly not	6.2	12.2
	Rather not	60.6	60.3
	Makes no difference	32.0	25.3
	Rather yes	0.4	1.3
	Certainly yes	0.8	0.9

Chi square analyses * p < 0.01, ** p < 0.001.

### Exploratory data analysis

To have a better understanding of this worsening of general image, we further explored underlying concepts that may help explain why the students’ image regarding older persons had worsened. To do so, exploratory principal component analyses were performed to identify underlying patterns in the data. Using PCA, the dominant patterns in the variables of the questionnaires resulted in the identification of five concepts that best reflect the themes used in the students’ appraisal of older persons. Concept scores were calculated on a scale of 1 to 7, the highest indicating worst possible image.

The first concept we called ‘personality traits’, as it is a combination of characteristics that are related to the personality of an older person, forming an individual's character (e.g. friendly, generous, and optimistic). The second is named ‘comportment’, as it is based on the way older persons conduct themselves towards others in response to a particular situation. Contrary to ‘personality traits’, ‘comportment’ is not considered part of an individual’s essential nature, rather a result of external stimulation. The third, ‘undesirable interaction’, encompasses negative responses from other people, making older persons unwanted or disliked. The opposite of this, ‘pleasurable interaction’, is the manner in which older persons behave in what can be seen as conducive and satisfactory contact with others. The fifth we called ‘habitual behaviour’ as it describes how older individuals interact with and maintain their living environment and daily living. See S1 Table for the variables included in each concept.

### Effect of intergroup contact on concepts

**Fig 1:**
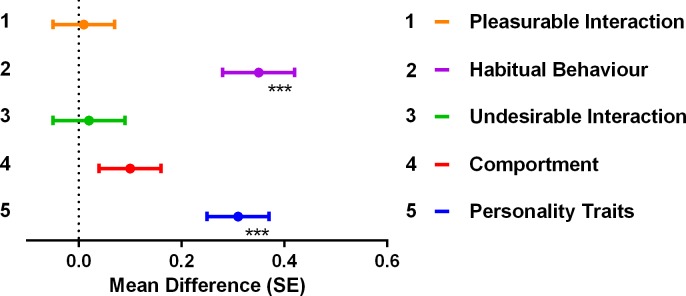
Effect of Intergroup Contact on Concepts. *** p < 0.001. A mean positive difference represents worsening image on concepts after intergroup contact.

Using the concepts identified from the principal component analysis; analyses of variance were performed to compare population means and standard deviations before and after contact on all identified concepts. Before contact, results indicated negative image towards older persons, the worst being the ‘comportment’ (mean 4.51, SD 0.63) and ‘pleasurable interaction’ (mean 3.68, SD 0.63). Fig 1 details how students’ image towards older people worsens considerably after intergroup contact. Significantly worsening were the concepts ‘personality traits’ (mean difference 0.31, SE 0.06, p < 0.001) and ‘habitual behaviour’ (mean difference 0.35, SE 0.07, p < 0.001). Worsening scores on ‘personality traits’ in this case means that students tend to view older persons as even more unfriendly, distrustful, unhappy, sloppy, selfish, unpleasant, dissatisfied, discouraged, pessimistic, and uncooperative after intergroup contact. As for ‘habitual behaviour’, after intergroup contact the students perceive older persons as even more unclean and untidy in their appearance and felt that they tended to neglect their house and let it become unattractive. It is also worth noting that in these two particular concepts not only did the scores worsen significantly, but the differences between before and after were also the highest compared to the other concepts identified.

**Fig 2:**
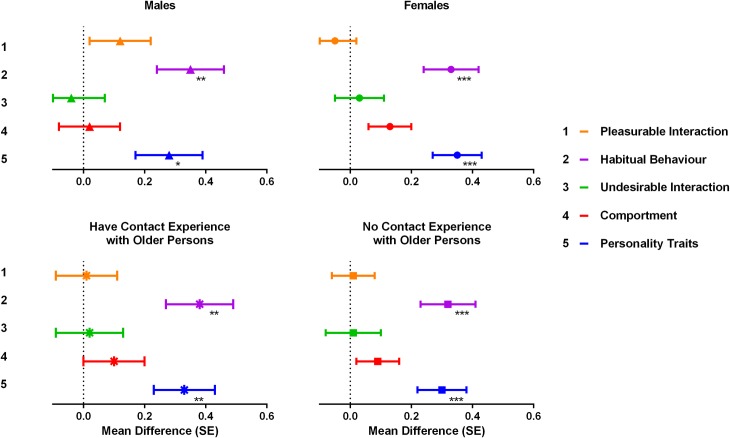
Effect of Intergroup Contact on Concepts Stratified by Gender and Contact Experience of the Students. * p < 0.05, ** p < 0.01, *** p < 0.001. A mean positive difference represents worsening image on concepts after intergroup contact.

As seen in Fig 2, these significant differences remain more or less the same when the student’s gender was taken into account (for males p = 0.011 and p = 0.002 respectively, for females p < 0.001 for both). In a further analysis, we categorized students into those who already had previous contact experience and those who did not have any contact with older individuals before the internship. Students were included in the first group if they answered yes to at least one question regarding living in a house with older persons; having had a previous internship; working; or having volunteer experience with older persons. When comparing between those who had previous contact experience and those without, patterns of worsening image did not alter the results (both groups p < 0.001).

## Discussion

This study investigates the triad of knowledge, contact, and attitude by examining the image first year medical students hold towards older persons before and after a care internship, and which concepts play a role in their image of older people. Findings indicated that before contact, they already held a negative image of older people. After intergroup contact with older persons and with the knowledge gained from an introductory seminar, there appears to be an overall tendency towards a further worsening of their image. This declining image applies to all sub-groups irrespective of gender and previous contact experience with older persons. Further explorations were performed in search of underlying concepts that may help explain this worsening image.

### Before contact, negative image towards older people exists

From the beginning of care internship, their image of older persons consisted of primarily negative designations. This is as expected, as a recent study found that age stereotypes became significantly worse over the span of two centuries[19]. All of this is epitomized in the fact that most would rather not work with older people both before and after the care internship. The results suggest that there is a confirmation of already negative images held towards older people during the internship. This may be explained by their demographic characteristics. The students involved in this study are relatively young and studies have shown that in general, ageism is more pronounced in younger individuals[20], because it allows younger people to distance themselves from older people in a positive way[21]. The majority of students did not have extensive previous contact experience with older persons. Further, their knowledge is limited as they were only given an introductory lecture and did not have any substantial training, considering that the care internship starts in their very first month of study of their first year.

### After contact, the negative image worsened

The explanation for their worsening attitude after contact may be attributed to the setting of this intergroup contact. In theory, people observe behaviour of other people based on the social roles they have, which may not fully reflect the actual personality of the person being observed[22]. In their care internship, the students see primarily institutionalized older persons who are in ill-health, which may confirm already negative images held of older persons. It is more than just old age, but more importantly the physical, debilitating aspect of the ageing process that is found to be viewed negatively by younger people[21]. Furthermore, these young students may use their own reference frame to evaluate what a good life should be, since stereotypes are formed as a reflection of our observations, expectations, and knowledge[23]. Perhaps these older people represent the antithesis of their expectations. The internship may highlight what they do not desire as people tend to create psychological barriers using their societal perspective and self-assurance to deal with the realization of the inevitable end of their life[21]. Whereas in reality, people tend to adjust their expectations and references of what a good life is as they grow older[24,25]. Intergroup anxiety, the discomfort that arises when interacting with another group, may also be a factor, as it can neutralize the positive effects of contact in improving image[26]. It is especially more likely to occur when there has been little or no previous contact or when negative stereotypes exist, as is the case in our study[27].

### Concepts identified from exploratory analysis

To figure out the possible explanations for this worsening general image, we explored the underlying concepts appraising older people captured in the two questionnaires used in the study. Here we identified five exploratory concepts that reflected the student’s appraisal of older people covering various domains. These findings are consistent with previous literature, which noted that people’s image towards older people are multidimensional, including positive and negative components[22]. It is essential to investigate this multidimensional nature because it allows us to examine the variety of beliefs and attitudes which can be inconsistent from time to time, further highlighting the complexity of changing attitudes toward older people[22]. All of the exploratory concepts identified in our study were in accordance with negative and positive clusters of ageing stereotypes identified by Smith and Boland[28]. Especially so for ‘personality traits’ and ‘undesirable interaction’, possibly because these two concepts are the most distinct and thus easily identified. Studies have found that older people have been constantly perceived as high on warmth (affectionate, friendly, good natured, kind, and trustworthy) and low on competence (independent, industrious, intelligent, productive, self-confident, and smart)[22,29–31], which respectively corresponds to our ‘personality traits’ and ‘comportment’ concepts. Kite et al. [22] also identified ‘age-related stereotypes’, which to some extent covered items from our ‘habitual behaviour’, ‘pleasurable interaction’, and ‘undesirable interaction’ concepts.

### Two concepts deteriorated significantly

Two specific concepts deteriorated significantly, namely how students viewed ‘personality traits’ of older persons and in their image of ‘habitual behaviour’. The declining image in these two aspects specifically may be largely caused by the instrumental nature of their care tasks, as elements in these concepts might be the most visible and readily observed and experienced during their care internships with frail older people. The fact that the three other concepts identified include characteristics that require some form of personal interaction is also indicative for our idea that the results can be explained by the limited actual close, personal contact during the internship and the nature of care tasks. It may also be that their expectations were more positive on the concepts ‘personality traits’ and ‘habitual behaviour’, as both scored relatively positive before the care internship compared to the other concepts.

### Recommendations based on earlier studies

One of the three known mediators in reducing prejudice is by enhancing knowledge[12], and it seems that the knowledge gained from the introductory lecture provided was not enough to create a more positive image of older people. Lack of knowledge about ageing could play an important role[32], so we would recommend more in-depth education before students participate in care internships. The other two mediators are reducing anxiety towards intergroup contact and enhancing empathy by facilitating situations in which one takes the perspective of the other group]12]. In order for these two mediators to work in diminishing negative stereotypes, positive and personal contact in an appropriate context is highly critical and should be fostered by the contact environment. In this care internship, such a context for instance through common goals, intergroup cooperation, and personal interaction were limited and this might explain the worsening results on ‘habitual behaviour’ and ‘personality traits’ concepts. Also, the contact developed between students and older persons might be of a rather superficial nature as students are preoccupied with instrumental care tasks, which do not allow for in-depth personal interaction. In these circumstances, following Allport, contact would not reduce prejudice, as the lack of personal experience with the out-group members may increase prejudice towards the other group[9]. The implication of this instrumental contact would be that students tend to behave in a defensive manner that leads to an unpleasant rather than a pleasant interaction and by extension their image towards older persons deteriorates. As a result, this care internship had the negative effect of unintentionally reinforcing ageism. With older persons being the majority of future patients, eliminating ageism should be included as one of the primary goals for these kinds of care internships, as it plays a significant role in shaping young medical students’ image towards older persons and their future career choices.

Quality of contact is the strongest predictor of attitudes towards older people among undergraduate students[33,34], as high quality contact fills out missing information, for instance by positive attributes, and can eliminate negative stereotypes[35]. If the quality of contact is high i.e. in-depth, then this may lead to improved attitudes, as shown by a trial among students visiting geriatric patients at home[36]. Furthermore, more long-term and frequent high quality contact seems to reduce prejudice[35]. Other than quality of contact, positive results may be further amplified if the students familiarize themselves with community dwelling older people. For instance, a Swedish study found that health professionals who performed preventive home visits gained a positive image towards older people[37]. This study highlights the importance of familiarizing students first with healthy community dwelling older persons, before assigning them to be involved with institutionalized older patients who require special attention and care. Besides the quality of contact and contact setting, context of contact is important as it helps create the nature of communication and enhance a sense of commonality and empathy, which is essential for positive contact[38,39]. Rather than enforcing impersonal and instrumental manual work, students and older persons should first work together on constructive activities that require intergroup cooperation and allow for common goals[40]. Members of these two age groups should be encouraged to disclose personal information to one another, hence filling in missing information therewith enhancing knowledge, reducing anxiety, and allowing integration of new perspectives and create empathy with one another. This enables opportunities to develop close interpersonal relationships, thus reducing prejudice, before they delve into the care tasks needed. A care internship at a later stage in their studies, after this kind of contact has already taken place, is therefore recommendable.

### Strengths

It has to be noted that studies which specifically investigate student’s attitudes toward older persons are scarce[13]. The strength of current study is that the intergroup contact took place in a real life setting with the most vulnerable older persons. Furthermore, previous contact experience with older people was also investigated. Finally, response rate is relatively high for a survey study.

### Limitations

There were several limitations that could have affected outcomes of this study. One major limitation in this study is that in our effort to ensure anonymity among the participating students, we did not link individual data of the before and after questionnaires. In doing so, the extent of overlap of persons across the two surveys is not known, as not all participating students completed both surveys and firstly these non-responses could lead to systematic bias. We would expect such bias to occur when for instance certain students did not feel motivated to fill in the questionnaire twice. Reasons for this may have been that they were unmotivated for the topic, they did not have the time or energy, or they were merely absent because of other activities or illness. Unfortunately, we did not have the necessary variables to perform sensitivity analyses to look into this. When comparing the outcomes on other variables (such as gender and age distribution), though, we did not find any significant differences. We would, moreover, argue that our response rate was quite high for a survey study; 80% and 77% respectively. Therefore, we would expect that the majority of participating students have responded both the before and after questionnaire while the lesser proportion of students have not responded twice. Secondly, a related issue is that, as there is no way to link the data across assessments, our analyses claim statistical significance using an independent group t-test under the assumption of independence. However, we do not know who dropped out and who did not. An independent group t-test is known to be more conservative than using the appropriate paired t-test or random effects analysis that assumes each pair to be positively correlated across time. Empirical evidence for such a correlation however, is not computable from the current data and the tests that have been applied are expected to err in a conservative manner, but still provide significant differences. In the unpaired analyses that we have performed, the confidence intervals and standard errors are normally larger than in paired analyses. Thus, if we could have linked the individual data and perform the paired analyses, we would expect these intervals and standard errors to become smaller. Nevertheless, given the use of an independent group t-test, this means that our results should be interpreted with caution.

In our follow-up study, we performed in-depth qualitative analysis to gain better insight into the students’ experiences. This study did not delve further into the frequency of previous contact or the experienced quality of contact. Another possible limitation could be that two weeks of this kind of contact is too short for intergroup contact to achieve the desired effects. Given that, however, these internships are normally relatively brief in medical schools and were quite intense consisting of full day immersions, this study does allow for assessing the impact of these internships on the image younger students hold towards older persons in a natural setting.

## Conclusion

Findings from this study indicated a tendency towards worsening image after intergroup contact during a care internship with older persons, especially with regard to ‘personality traits’ of older persons and their ‘habitual behaviour’. After the care internship, there seems to be a confirmation of the already negative image of older people these students hold. Hence, it has the negative effect of unintentionally reinforcing ageism. This might be due to inadequate contact depth and quality within an unsupportive context in an unsuitable contact setting.

Interventions should be designed in ways that allow for high quality contact. Medical schools should carefully consider this when developing their practical immersions, since if we wish to provide quality of care in the future; we need to ensure that future medical practitioners do not worsen their views on their future patient population.

## Supporting Information

S1 Appendix Care Placement Survey Questionnaire. (DOCX)

S1 Table Five Concepts Identified from Exploratory Principal Component Analysis. (DOCX)

S2 Table Component Loadings for Aging Semantic Differential Questionnaire. (DOCX)

S3 Table Component Loadings for Attitudes toward Old People Questionnaire. (DOCX)
