# Supplementary material for: When Contact Is Not Enough: Affecting First Year Medical Students’ Image towards Older Persons
Source: PLoS One. 2017 Jan 20;12(1):e0169977. doi: 10.1371/journal.pone.0169977 (PMC5249097; doi:10.1371/journal.pone.0169977)
Supplement: S1 Appendix — (DOCX) [file pone.0169977.s001.docx]

# **S1 Appendix. Care Placement Survey Questionnaire.**

To guarantee privacy, accompanying questionnaires are completed anonymously. This also makes it easier to answer the questions honestly. Still, you are prompted to enter a four-digit PIN (e.g. the last four digits of a telephone number; no date of birth!). To have better grasp on which factors influence the response pattern, below are some general questions. Please mark multiple answers by circling the correct answer.

1. Code number (four digits): ................. (do not fill)
2. Age (in years): .....................................
3. Gender: female male
4. Education:
5. Have you already done an internship with older persons in your training?

yes no

1. Have you ever worked as a volunteer with older persons?

yes no

1. How many of your grandparents are still alive? .......................................
2. How old are they? .....................................................................................
3. How many of your great-grandparents are still alive? ..............................
4. How old are they? .................................................. ..................................
5. Do you live in a house where older persons live?

yes no

1. Do you have a family member, friend, or neighbour with dementia?

yes no

1. Do you think that you as a health care provider or counsellor will find as much satisfaction in the care of older people as with younger patients? yes no
2. Do you have experience working as a carer? *

yes no

1. If you have experience as a caregiver, for whom?
    family friends neighbours extended family
2. Does anyone in your family provide care? yes no

17. As part of your medical training, in which health sector would you prefer to work during your internship? (circle one option, even if you are not sure)?
 paediatrics psychiatry homecare general hospital nursing home
18. Would you like to work in a practice where most patients are older than 65 years after your training (circle one option, even if you are not sure)?
 certainly not rather not makes no difference rather yes certainly yes

* Carer is a collective name for non-professional, voluntary provision of non-medical assistance and service to people asking for help (friends, neighbours, family, family members) with whom the provider has an initial socio-affective bond.
