# Supplementary material for: When Contact Is Not Enough: Affecting First Year Medical Students’ Image towards Older Persons
Source: PLoS One. 2017 Jan 20;12(1):e0169977. doi: 10.1371/journal.pone.0169977 (PMC5249097; doi:10.1371/journal.pone.0169977)
Supplement: S1 Table — (DOCX) [file pone.0169977.s002.docx]

# **S1 Table. Five Concepts Identified from Exploratory Principal Component Analysis**

| **‘personality traits’** | **‘comportment’** | **‘habitual behaviour’** | **‘pleasurable interaction’** | **‘undesirable interaction’** |
| --- | --- | --- | --- | --- |
| Friendly / unfriendly  Trusting / distrustful  Happy / unhappy  Neatly / sloppy  Generous / selfish  Nice / unpleasant  Satisfied / dissatisfied  Hopeful / discouraged  Optimistic / pessimistic  Cooperative / not cooperative | Being active / passive  Industrious / inactive  Progressive / out-dated  Independent / dependent  Productive / unproductive  Strong / weak  Beautiful / ugly  Freethinking / conservative  Exciting / boring  Healthy / unhealthy  Expectantly / resigned  Flexible / not flexible | Clean and tidy in their appearance  An area is neat when significant number of older persons live there  Tend to keep their house neat and appealing  Should be more worried about their appearance as they are too dirty  Tend to neglect their house and let it become unattractive  It is advisable not to have too many older persons to keep a neighbourhood tidy. | As easy to understand as younger adults  Can adapt if the situation requires  Should have more power in business and politics  Cheerful, pleasant, good mood  Rarely heard complaining about the behaviour of young generation  Inclined to deal with their own affairs and only give advice when asked  It is relaxing to be with most older persons. | Most of them look similar  Nosy and give unsolicited advice  Need to get rid of annoying flaws to be liked  Bore others by insisting on talking about the good old days  Constantly complain about the behaviour of younger generation  Make you feel uncomfortable  Irritable, grumpy, and unsociable  Too much power in business and politics |
