# Supplementary material for: When Contact Is Not Enough: Affecting First Year Medical Students’ Image towards Older Persons
Source: PLoS One. 2017 Jan 20;12(1):e0169977. doi: 10.1371/journal.pone.0169977 (PMC5249097; doi:10.1371/journal.pone.0169977)
Supplement: S2 Table — (DOCX) [file pone.0169977.s003.docx]

# **S2 Table. Component Loadings for Aging Semantic Differential Questionnaire**

|  | | |
| --- | --- | --- |
|  | Component | |
|  | 1 | 2 |
| ASD.21_friendly / unfriendly | .744 | .170 |
| ASD.23_trusting / distrustful | .677 | .211 |
| ASD.28_nice / unpleasant | .670 | .366 |
| ASD.20_happy / unhappy | .661 | .324 |
| ASD.15_satisfied / dissatisfied | .651 | .349 |
| ASD.5_generous / selfish | .627 | .260 |
| ASD.22_neatly / sloppy | .626 | .227 |
| ASD.18_hopeful / discouraged | .623 | .430 |
| ASD.14_optimistic / pessimistic | .609 | .405 |
| ASD.13_cooperative / not cooperative | .472 | .403 |
| ASD.27_tolerant / intolerant | .373 | .196 |
| ASD.29_everyday / eccentric | .313 | .227 |
| ASD.30_aggressive / defensive | .312 | -.035 |
| ASD.24_financially independent / financially dependent | .277 | .007 |
| ASD.8_safe / unsafe | .275 | .225 |
| ASD.11_active / passive | .262 | .751 |
| ASD.7_industrious / inactive | .247 | .699 |
| ASD.6_productive / unproductive | .285 | .628 |
| ASD.3_independent / dependent | .200 | .594 |
| ASD.9_strong / weak | .142 | .590 |
| ASD.1_progressive / out-dated | .224 | .585 |
| ASD.12_beautiful / ugly | .138 | .580 |
| ASD.10_healthy / unhealthy | .340 | .517 |
| ASD.31_exciting / boring | .205 | .488 |
| ASD.25_freethinking / conservative | .429 | .450 |
| ASD.16_expectantly / resigned | .058 | .442 |
| ASD.17_flexible / not flexible | .274 | .404 |
| ASD.32_resolute / indecisive | .258 | .382 |
| ASD.26_certainly / uncertain | .294 | .338 |
| ASD.19_organized / disorganized | .334 | .334 |
| ASD.2_consistent / inconsistent | .205 | .225 |
| ASD.4_rich / poor | .153 | .162 |
