# Supplementary material for: When Contact Is Not Enough: Affecting First Year Medical Students’ Image towards Older Persons
Source: PLoS One. 2017 Jan 20;12(1):e0169977. doi: 10.1371/journal.pone.0169977 (PMC5249097; doi:10.1371/journal.pone.0169977)
Supplement: S3 Table — (DOCX) [file pone.0169977.s004.docx]

# **S3 Table. Component Loadings for Attitudes toward Old People Questionnaire**

|  | Component | | |
| --- | --- | --- | --- |
|  | 1 | 2 | 3 |
| AOP.12_nosy and gives unsolicited advice | .603 | -.017 | -.225 |
| AOP.17_most of them look similar | .591 | -.001 | -.084 |
| AOP.13_need to get rid of annoying flaws to be liked | .591 | .174 | -.102 |
| AOP.21_bore others by insisting on talking about the good old days | .560 | .251 | -.192 |
| AOP.9_make you feel uncomfortable | .537 | .284 | -.116 |
| AOP.34_irritable. grumpy. and unsociable | .530 | .301 | -.376 |
| AOP.29_constantly complain about the behaviour of younger generation | .498 | -.167 | -.330 |
| AOP.8_too much power in business and politics | .412 | .198 | .015 |
| AOP.4_most wanted to stop working if pension sufficient and children could support | .398 | .190 | .023 |
| AOP.28_most differ very much from each other | -.372 | -.090 | .030 |
| AOP.32_foolish to claim wisdom comes with age | .359 | .182 | -.258 |
| AOP.6_most of them are different. difficult to understand | .295 | .218 | -.281 |
| AOP.1_better if they live with people of their own age | .191 | .183 | -.143 |
| AOP.24_tend their house neat and keep it appealing | -.380 | -.587 | .236 |
| AOP.15_an area is neat when significant number of older persons live there | .060 | -.567 | .086 |
| AOP.20_clean and tidy in their appearance | -.262 | -.547 | .350 |
| AOP.18_should be more worried about their appearance as they are too dirty | .422 | .527 | -.158 |
| AOP.5_tend to neglect their house and let it become unattractive | .490 | .501 | -.170 |
| AOP.30_it is advisable not to have too many older persons to keep a neighbourhood tidy | .392 | .494 | -.015 |
| AOP.14_most would rather continue work than depend on someone | -.129 | -.352 | .107 |
| AOP.11_interesting and entertaining qualities are stories of past experiences | -.107 | -.318 | .011 |
| AOP.25_most have high demands in terms of love and reassurance | -.075 | -.249 | .042 |
| AOP.3_can adapt if the situation requires | -.142 | -.109 | .556 |
| AOP.2_as easy to understand as younger adults | .027 | -.194 | .513 |
| AOP.22_cheerful. pleasant. good mood | -.198 | -.326 | .511 |
| AOP.23_rarely heard complaining about the behaviour of young generation | -.169 | .287 | .481 |
| AOP.33_it is relaxing to be with most older persons | -.370 | -.193 | .451 |
| AOP.19_should have more power in business and politics | -.096 | .037 | .426 |
| AOP.26_inclined to deal with their own affairs and only give advice when asked | .050 | .315 | .378 |
| AOP.16_most are rust set in their ways and cannot change | .333 | -.218 | -.366 |
| AOP.7_people became wiser with the coming of age | -.141 | -.217 | .355 |
| AOP.31_most have no more love and attention than any other | .217 | .016 | .355 |
| AOP.10_better if older persons live in buildings where younger adults live as well | -.129 | -.236 | .304 |
| AOP.27_have the same flaws as everyone else | -.196 | -.020 | .266 |
